# Black fungi and ants: a genomic comparison of species inhabiting carton nests versus domatia

**DOI:** 10.1186/s43008-022-00091-5

**Published:** 2022-03-07

**Authors:** Yu Quan, Nickolas Menezes da Silva, Bruna Jacomel Favoreto de Souza Lima, Sybren de Hoog, Vania Aparecida Vicente, Veronika Mayer, Yingqian Kang, Dongmei Shi

**Affiliations:** 1 Center of Expertise in Mycology of Radboud University Medical Center/Canisius Wilhelmina Hospital, Nijmegen, The Netherlands; 2 Department of Dermatology and Laboratory of Medical Mycology, Jining No. 1 People’s Hospital, Shandong, China; 3 Key Laboratory of Environmental Pollution Monitoring and Disease Control, Ministry of Education of Guizhou and Guizhou Talent Base for Microbiology and Human Health, School of Basic Medical Sciences, Guizhou Medical University, Guiyang, China; 4 Guizhou Provincial Academician Workstation of Microbiology and Health, and Guizhou Academy of Tobacco Science, Guiyang, China; 5 Graduate Program in Microbiology, Parasitology and Pathology, Biological Sciences, Department of Basic Pathology, Federal University of Paraná, Curitiba, Brazil; 6 Department of Botany and Biodiversity Research, University of Vienna, Wien, Austria; 7 Graduate Program in Bioprocess Engineering and Biotechnology, Federal University of Paraná, Curitiba, Brazil

**Keywords:** Black fungi, Carton fungi, Chaetothyriales, Comparative genomics

## Abstract

Some members of Chaetothyriales, an order containing potential agents of opportunistic infections in humans, have a natural habitat in nests of tropical arboreal ants. In these black fungi, two types of ant symbiosis are known, i.e. occurrence in domatia inside living plants, or as components of carton constructions made of ant-chewed plant tissue. In order to explain differences between strains from these types of association, we sequenced and annotated genomes of two newly described carton species, *Incumbomyces lentus* and *Incumbomyces delicatus*, and compared these with genomes of four domatia species and related Chaetothyriales. General genomic characteristics, CYP genes, carbohydrate-active enzymes (CAZymes), secondary metabolism, and sex-related genes were included in the study.

**Supplementary Information:**

The online version contains supplementary material available at 10.1186/s43008-022-00091-5.

## Introduction

Ants (Arthropoda: Formicidae) are ubiquitous in nearly all climate zones worldwide and play important roles in many ecosystems. The sum of ant bodies has been estimated to account for 15–20% of terrestrial animal biomass (Schultz 2000). Interactions between ants and fungi were already recorded more than 100 years ago (Bailey 1920; Huxley 1978; Miehe 1911). These not only concern the Attine-fungi mutualistic symbioses in general in fungus gardens (Mueller et al. 2018), but a wide diversity of less pronounced interactions occur (Biedermann and Vega 2020; Moreau 2020). Among the black fungi, *Cladosporium myrmecophilum* is a classical colonizer of debris in carton nests of *Lasius fuliginosus* (Schlick-Steiner et al. 2008). Fungi identified with sequence data as belonging to the order Chaetothyriales were found a decade ago, when phialophora-like species were recurrently isolated from ants and their constructions (Little and Currie 2007; Defossez et al. 2009; Mayer et al. 2018). Many of these fungi seem to have ant-association as their main habitat choice, and many are new to science. Given the wide distribution, biodiversity and nesting activities of ant species, the fungi associated with these arthropods might also be quite diverse. The discovery and full description of these new species will have a profound impact on the current classification of Chaetothyriales.

The order Chaetothyriales (Ascomycota) is particularly known through the black yeasts and filamentous relatives that cause opportunistic infections in humans. The species in this order tend to be abundant in extreme or tannin-rich natural habitats. The best-known species, however, are the ones that are found in domesticated environments that are rich in creosote or toxic hydrocarbons (Döğen et al. 2013) or in habitats with high temperature and/or poor in nutrients (Gostinčar et al. 2012). This extremotolerance has been hypothesized to contribute to invasive abilities of sterile sites of the vertebrate body (Quan et al. 2020a, b). As ants produce and communicate with ketones and low-molecular hydrocarbons (Di Mauro et al. 2015) which also function as antimicrobial compounds in ant nests and constructions, the ants might have played a role in early evolution of extremophily observed in Chaetothyriales. While nine clades of the order have been redefined to family level (Quan et al. 2020a, b), the majority of species with close vicinity to ants, i.e. those residing in their nesting space provided by their host-plant (= domatia), clustered in a single, as yet poorly defined clade, which might deserve family status. Some additional domatia-associated species are found in the Trichomeriaceae. Species associated with carton exposed to the environment seem less specialized and are scattered in three families, i.e. Cyphellophoracere, Herpotrichiellaceae, and Trichomeriaceae (Voglmayr et al. 2011; Vasse et al. 2017). Morphologically, *Incumbomyces* species, as other carton strains, lack conidiation in culture, while domatia-fungi show a consistent type of sympodial propagation with mucous conidia (Voglmayr et al. 2011; Quan et al. 2020a, b). The morphological differences might be related to vectors of dispersal e.g. with ant bodies.

The deviating morphology and phylogenies of carton- *versus* domatia-associated black fungi suggests that Chaetothyriales have gone through different types of evolution with the ant as driver. Domatia are modified leaves, stems or roots that provide cavities occupied by ants, offered by about 680 species of tropical vascular plants. Once inhabited by ants, the domatia contain dark patches from which black fungi can be isolated (Nepel et al. 2016). Recent data show that colony-founding queens start growing chaetothyrialean fungi in the domatia already before they lay their eggs, and that the queens do not feed on fungal material themselves but feed it to the larvae (Mayer et al. 2018). The second type of association of ants and Chaetothyriales is referred to as carton nesting. Nests consist of chewed plant material and have a cardboard-like appearance. Fungal hyphae are abundantly found in the walls of nests and galleries and strengthen the constructions. The main function of the carton structure is to increase the space of the nest and to enhance defense strategies (Vasse et al. 2017). The fungi in these structures do not serve as food, but increase stability of the nests (Hölldobler and Wilson 1990).

Genome analysis and comparison is a widely used biotechnology and may provide clues towards understanding of microbial ecology. At present, 45 genomes of Chaetothyriales have been published. Teixeira et al. (2017) analyzed 23 genomes of Chaetothyriales and provided a detailed comparative analysis, revealing genes related to protein degradation, carbohydrate-active enzymes (CAZymes), melanin synthesis and secondary metabolism. Moreno et al. (2019) was the first to analyze genomes of Chaetothyriales from ant domatia. The domatia-associated species were found to have remarkably small genomes, low amounts of protein-coding genes, and a high degree of repetitive elements. In addition, the proportion of biosynthetic clusters involved in the production of secondary metabolites and potential antibacterial activities were overrepresented. Attili-Angelis et al. (2014) described some species from ant bodies rather than from nesting material; these genomes represent members of Cyphellophoraceae.

Two novel genomes in the present study were derived from CBS 128958 and CBS 129047, recently described as types of *Incumbomyces lentus* and *I. delicatus*, respectively, in the family Trichomeriaceae. Both originated from ant carton structures of ant species in tropical Southeast Asia structures (Voglmayr et al. 2011). Although carton fungi and domatia fungi both belong to Chaetothyriales, their positions within the order are markedly different. Habitat conditions within domatia are balanced, whereas carton structures are subjected to fluctuating environmental conditions. The difference in habitat choices of the black fungi may to have had profound evolutionary consequences. The aim of the present paper is to compare some parameters which may have played a role in these differential phylogenies.

## Materials and methods

### Strains and sequencing

Genomic DNA of *Incumbomyces lentus* CBS 128958, isolated from carton of a *Monomorium* sp. ant nest in Malaysia and *I. delicatus* CBS 129047, isolated from carton of a *Crematogaster* sp. ant nest in Thailand (Quan et al. 2020a, b) was extracted from cell pellets harvested from cultures incubated for 14 days at 28 °C with Fungi DNA Kit (Omega Bio-Tek, Norcross, GA, U.S.A.) according to the manufacturer’s instructions. DNA concentration was quantified using a TBS-380 fluorometer (Turner BioSystems, Sunnyvale, CA, U.S.A.). High qualified DNA samples (OD_260/280_ = 1.8–2.0, > 6 µg) were utilized to construct a fragment library with 400 bp insert size. Sequencing was performed at Biozeron Biotechnology Company (Shanghai, China) on Illumina Hiseq and Pacific Bioscience platforms. Information of species included in the study is provided in Additional file 1: Table S1.

### Alignment and phylogenetic analysis

The combined sequences of ITS and LSU of Chaetothyriales species in this study were obtained from NCBI and edited using BioEdit v7.2 (Hall 1999). Alignments were made by Mafft v7 (http://mafft.cbrc.jp/) and optimized manually using Mega v7.2 (Kumar et al. 2012) and BioEdit v7.2. Missing data for partial or complete sequences in some taxa were coded as ‘missing’ (Wiens 2006). To address the phylogenetic relationships among taxa, Maximum Likelihood (ML) was used. Species of *Melanina* were taken as outgroups. The ML tree was obtained using RAxML-VI-HPC as implemented on the Cipres portal web server (http://www.phylo.org/). The tree was edited using Treeview v1.6.6 and completed with Adobe Illustrator CS v5.

### Genome assembly, repeat identification

Raw sequencing data was generated by Illumina base calling software Casava v1.8.2 (http://support.illumina.com) with default settings. Contaminations of adaptors and primers were identified by Trimmomatic (http://www.usadellab.org) with default parameters. Completeness of genomes was verified using ABySS (http://www.bcgsc.ca) with multiple-Kmer parameters. GapCloser software (https://sourceforge.net) was subsequently applied to fill remaining local gaps and correct SNPs for the final assembly. Repeatmasker (http://www.repeatmasker.org/) with default parameters was used to identify genome repeat information (Stanke and Waack 2003). The genome sequences were submitted to NCBI with the accession numbers JACJVS000000000 (*I. delicatus*) and JACJVT000000000 (*I. lentus*).

### Gene prediction and functional annotation

Genes of the studied isolates were predicted by ab initio prediction methods to obtain gene models for the organisms. Gene models were identified using Augustus (Stanke and Waack 2003). Cytochrome P450 monooxygenases (CYPs) were identified and annotated though CYPminer, which is an automated computational pipeline for identification, classification, and downstream analyses of CYPs at the genome level (Kweon et al. 2020). According to the International P450 Nomenclatural Committee, a sequence identity of > 40% is regarded as the same family, and > 50% is regarded as the same subfamily. Carbohydrate-Active Enzymes (CAZymes) were identified using the dbCAN2 web server (Zhang et al. 2018). To identify and classify clusters of genes involved in the production of secondary metabolites, the genomes of the carton-associated species were mined by the antiSMASH web server, fungal version 5.0 (https://fungismash.secondarymetabolites.org; Blin et al. 2019). The mating type loci were characterized by homology to the *MAT1-1* and *MAT1-2* reference sequences previously described in Chaetothyriales.

## Results

### Phylogeny

**Fig. 1:**
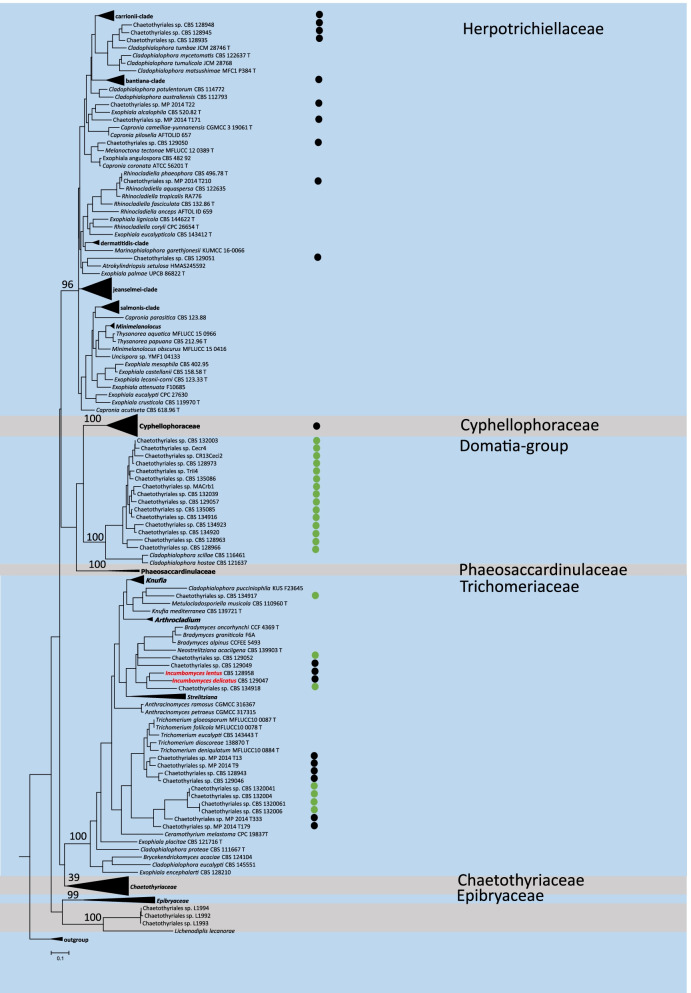
Phylogenetic tree of Chaetothyriales based on ITS and LSU sequences, obtained by maximum likelihood. Four *Melanina* strains were used as outgroup. Green dots represent domatia fungi and black dots represent carton fungi

The phylogenetic tree contains a total of 255 sequences including four of *Melanina* as outgroup (Additional file 2: Table S2). Two carton species, four domatia strains and 249 remaining Chaetothyriales species were analyzed. Throughout the tree, eight clades described at family level were recognized. Bootstrap values of each family are showed in Fig. 1. Carton strains are scattered throughout the tree, clustering in three families: Herpotrichiellaceae, Cyphellophoraceae and Trichomeriaceae. Most domatia strains clustered in a single clade together with two *Cladophialophora* species. A few domatia strains clustered in the family Trichomeriaceae, close to carton strains which are rather commonly found in the same family.

### Genome assembly and gene prediction

**Fig. 2:**
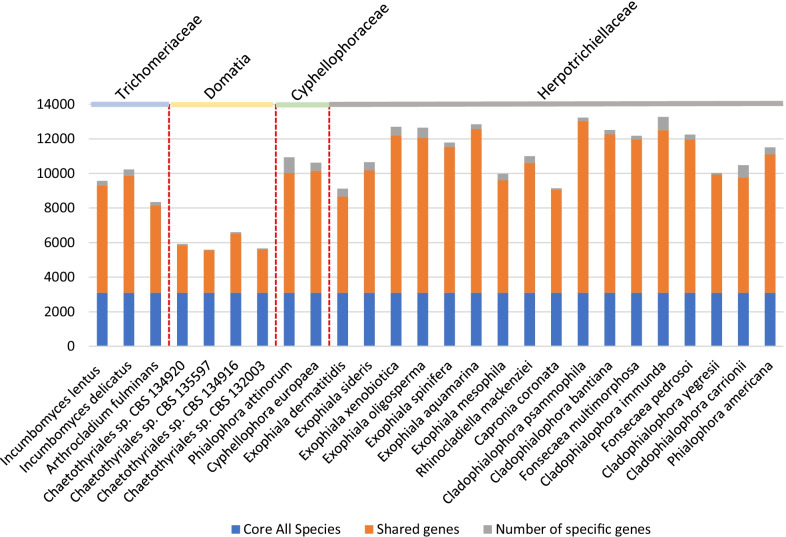
Orthology classes in ant-associated fungi and other Chaetothyriales fungi: core genes found in all genomes are shown in blue, shared genes present in more than one but not all genomes in orange and genes that were unique to only one of the analyzed genomes in grey

Genome sequences of two *Incumbomyces* species originating from carton of ant nests were determined by Illumina and Pacific Bioscience and de novo assembled. After cleaning, a total of 27.1 Mb and 31.5 Mb high quality reads were generated for the strains CBS 128958 and CBS 129047, *I. lentus* and *I. delicatus*, respectively. GC contents were 50.78% (*I. lentus*) and 49.37% (*I. delicatus*), which is similar to most other black yeast species (Teixeira et al. 2017). Protein-coding gene compositions were determined by ab initio gene prediction methods. The highest gene count of 9530 genes was found in *I. delicatus*, which also had the largest genome of all ant-associated species investigated (31.5 Mb), whereas the domatia-associated strain CBS 134916 comprised only 6719 genes (Moreno et al. 2019). *I. lentus* was intermediate in having 8868 genes, similar to the species *Arthrocladium fulminans* (8534 genes), which belongs to Trichomeriaceae, i.e., the same family as carton fungi in *Incumbomyces*. Both average gene lengths of carton fungi (in bp) were similar, i.e., 1645 and 1637 bp. The number of tRNAs were different between the two studied strains. Numbers of tRNAs of carton strains (143–208) were much higher than those in domatia-associated strains (37–76). The contents of repetitive elements in *I. lentus* (CBS 128958) and *I. delicatus* (CBS 129047) were 1.75% and 3.41%, respectively, which was on average significantly higher than in other thus far sequenced species of Chaetothytiales without ant-related ecology (ranging from 0.03 to 5.2%; Teixeira et al. 2017). In *Arthrocladium fulminans,* the repetitive elements comprised 1.75%, while in domatia fungi percentages were very high, ranging from 4.09 to 16.32%. All basic information of carton species, domatia species and *Arthrocladium fulminans* is listed in Table 1. Using Orthovenn2, we determined the core gene clusters that were conserved in both carton and domatia fungi, and in other species of Chaetothyriales. A range from 5586 to 13,269 orthologous clusters was detected among the 26 studied isolates, including carton fungi and domatia fungi (Fig. 2, Table 2). This resulted in 3096 clusters per genome in the core set conserved in all isolates. Among these, the average number of unique genes was 314 existing in the two carton fungi, while the average unique genes in domatia species only was 67, and 188 clusters were represented in *A. fulminans*.

**Table 1:** Statistics of compared genomes of ant-associated species and *Arthrocladium* sequenced in this study and by Moreno et al. (2019)

Strain number	CBS 129047	CBS 128958	CBS 136243	CBS 134916	CBS 132003	CBS 134920	CBS 135597
Species	*I. delicatus*	*I. lentus*	*A. fulminans*	Chaetothyriales sp	Chaetothyriales sp	Chaetothyriales sp	Chaetothyriales sp
Isolation source	Ant-carton	Ant-carton	Human patient	Domatium	Domatium	Domatium	Domatium
Gemone size (Mbp)	31.5	27.1	27.22	26.3	22.8	20.6	20.6
%G + C content	49.38%	50%	51.82%	50.55	54.00	53.82	52.73
N50 length (bp)	4,869,129	3,707,458	1,671,613	60,287	312,613	164,768	570,188
Gene number	9530	8868	8534	6719	5812	5988	5689
Repetitive elements	3.41%	1.75%	1.57%	16.32%	4.09%	8.67%	8.43%
tRNAs	143	208	unknown	37	43	76	60
NCBI number	JACJVS000000000	JACJVT000000000	GCA_003614865.1	QQXO00000000	QRBJ00000000	QQSL00000000	QQXN00000000

**Table 2:** The number of orthology classes in ant-associated fungi and other Chaetothyriales fungi, including core genes, shared genes, unique gene

Species	Number of genes in orthogroups	Core all species	Shared genes	Number of specific genes
Incumbomyces lentus	9570	3096	6214	260
Incumbomyces delicatus	10,228	3096	6765	367
*Arthrocladium fulminans*	8346	3096	5062	188
Chaetothyriales sp. CBS 134,920	5916	3096	2752	68
Chaetothyriales sp. CBS 135,597	5586	3096	2454	36
Chaetothyriales sp. CBS 134,916	6605	3096	3411	98
Chaetothyriales sp. CBS 132,003	5662	3096	2501	65
*Phialophora attinorum*	10,935	3096	6926	913
*Cyphellophora europaea*	10,622	3096	7054	472
*Exophiala dermatitidis*	9117	3096	5560	461
*Exophiala sideris*	10,650	3096	7084	470
*Exophiala xenobiotica*	12,696	3096	9109	491
*Exophiala oligosperma*	12,642	3096	8954	592
*Exophiala spinifera*	11,783	3096	8421	266
*Exophiala aquamarina*	12,839	3096	9464	279
*Exophiala mesophila*	9977	3096	6511	370
*Rhinocladiella mackenziei*	10,997	3096	7516	385
*Capronia coronata*	9140	3096	5953	91
*Cladophialophora psammophila*	13,227	3096	9937	194
*Cladophialophora bantiana*	12,512	3096	9166	250
*Fonsecaea multimorphosa*	12,173	3096	8881	196
*Cladophialophora immunda*	13,269	3096	9409	764
*Fonsecaea pedrosoi*	12,245	3096	8867	282
*Cladophialophora yegresii*	10,023	3096	6832	95
*Cladophialophora carrionii*	10,478	3096	6679	703
*Phialophora americana*	11,507	3096	8009	402

### Carbohydrate-active enzymes (CAZymes)

CAZymes are responsible for the degradation, modification, and biosynthesis of carbohydrates and glycoconjugates (Cantarel et al. 2009). Based on amino-acid sequence and structure similarity, the CAZymes can be classified into five classes of enzyme activities and one associated module: glycoside hydrolases (GHs), glycosyl transferases (GTs), polysaccharide lyases (PLs), carbohydrate esterases (CEs), auxiliary activities (AAs), and the associated module carbohydrate-binding modules (CBMs) (Cantarel et al. 2009). The total numbers of genes in CAZymes families of *I. lentus* and *I. delicatus* are 288 and 293, respectively. This is higher than found in domatia fungi (ranging from 208 to 233), but comparable to members of Herpotrichiellaceae (ranging from 285 to 434) and *A. fulminans* in Trichomeriaceae (269) (Table 3).

**Table 3:** Composition of CAZymes in ant-associated and related chaetothyrialean fungi

Species	GH	GT	CE	AA	CBM	PL	Total
**Trichomeriaceae**							
*Incumbomyces lentus*	128	81	37	40	1	1	288
*Incumbomyces delicatus*	135	79	38	39	1	1	293
*Arthrocladium fulminans*	135	62	39	31	1	1	269
**Domatia**							
Chaetothyriales sp. CBS 134920	87	74	30	30	1	1	223
Chaetothyriales sp. CBS 135597	85	74	26	33	1	1	220
Chaetothyriales sp. CBS 134916	96	76	30	30	1	0	233
Chaetothyriales sp. CBS 132003	86	68	27	26	1	0	208
**Cyphellophoraceae**							
*Phialophora attinorum*	151	98	69	66	3	1	388
*Cyphellophora europaea*	173	96	62	72	3	1	407
*Exophiala dermatitidis*	118	87	39	37	2	2	285
**Herpotrichiellaceae**							
*Exophiala xenobiotica*	181	110	70	67	4	2	434
*Exophiala oligosperma*	161	123	73	61	3	0	421
*Exophiala spinifera*	146	106	71	50	2	1	376
*Exophiala aquamarina*	162	96	84	63	4	0	409
*Exophiala mesophila*	118	102	49	52	1	0	322
*Rhinocladiella mackenziei*	119	76	53	58	4	1	311
*Capronia coronata*	112	84	52	34	3	2	287
*Capronia epimyces*	104	89	61	44	1	0	299
*Cladophialophora psammophila*	141	91	78	77	3	0	390
*Cladophialophora bantiana*	143	90	66	70	3	0	372
*Fonsecaea multimorphosa*	140	90	68	77	2	0	377
*Cladophialophora immunda*	148	101	64	71	2	0	386
*Fonsecaea pedrosoi*	133	89	81	65	2	0	370
*Cladophialophora yegresii*	129	82	46	48	2	0	307
*Cladophialophora carrionii*	137	87	43	50	3	1	321
*Capronia semiimmersa*	140	94	51	54	3	0	342

GH: glycoside hydrolases, GT: glycosyl transferases, CE: carbohydrate esterases, AA: auxiliary activities, CBM: associated carbohydrate-binding modules, PL: polysaccharide lyases

Polysaccharide lyases (PLs) are a group of enzymes that cleave uronic acid-containing polysaccharide chains via a β-elimination mechanism to generate an unsaturated hexenuronic acid residue and a new reducing end. Both *Incumbomyces* carton strains possess the polysaccharide lyase subfamily 3 (PL3); the two domatia strains (CBS 135957 and CBS 134920) possess subfamily 4 (PL1), while two domatia strains (CBS 134916 and CBS 132003) were similar to most other Chaetothyriales, lacking pectinases; PL3 was so far detected only in *Capronia coronata* (Herpotrichiellaceae), a species originating from decorticated wood, and in *Cyphellophora attinorum* (Cyphellophoraceae), which was isolated from the cuticle of tropical ant gynes (Additional file 3: Table S3).

Glycoside hydrolases (GHs) are a widespread group of enzymes which hydrolyze the glycosidic bond between two or more carbohydrates or between a carbohydrate and a non-carbohydrate moiety. The number of GH families of the carton fungi are 128 and 135, more than that present in domatia fungi (85–96; Table 3). GH families that exist in carton fungi but are absent from domatia fungi are GH27, GH36, GH79, and GH92. GH 27 and GH 36 mainly associated with galactosidase-related enzymes. GH 79 associated with glucuronidase-related enzymes, and GH92 are mainly focused on mannosidase.

Glycosyltransferases (GTs) are enzymes that catalyze the transfer of sugar moieties from activated donor molecules to specific acceptor molecules, forming glycosidic bonds. On average, members of Herpotrichiellaceae contain somewhat larger numbers (76–123, av. 93.9) than remaining members of Chaetothyriales (62–98, av. 78.7). A similar difference was observed with carbohydrate esterases (CEs), members of Herpotrichiellaceae having 39–84 (av. 61.7), against 26–69 (av. 39.8) for remaining species. *Exophiala dermatitidis* was consistently exceptional in Herpotrichiellaceae, having genome size and gene numbers like *Incumbomyces* species (Table 3).

The AA class presently groups nine families of ligninolytic enzymes and 6 families of lytic polysaccharide mono-oxygenases. A notable difference was observed in the numbers of AA7 of carton fungi being 14, while only 3–7 AA7 were present in domatia fungi.

### Cytochrome p450 genes (CYPs)

Cytochrome p450 genes exist widely in all kingdoms and play important roles in primary and secondary metabolism, and in drug and xenobiotic resistance. Our study used CYPminer, an automatic tool that allows processing of large numbers of sequence data to identify and classify CYPs. The identification results of CYPminer are more detailed and abundant than with use of the previous database, the PFAM protein family database. Analyzing the domatia strain, CBS 135597, 51 families were identified using the CYPminer tool, while only 14 results were obtained using the PFAM database. In order to compare CYPs in the carton fungi under study, we analyzed two domatia strains and nine additional species of Chaetothyriales (Table 4). A total of 216 CYP genes clustering in 130 families and 118 subfamilies were identified in the two *Incumbomyces* carton species. The number of CYPs of two carton species is slightly lower than that of two domatia strains, namely 105 (CBS 128958) and 111 (CBS 128047) in *Incumbomyces*, *vs.* 139 (CBS 135597) and 128 (CBS 134916) in domatia strains. The number of CYPs genes in compared members of Trichomeriaceae and Herpotrichiellaceae ranged from 117 (*Arthroderma fulminans*) to 322 (*Cladophialophora psammophila*). This result indicates that the CYP genes have significantly expanded in members of Herpotrichiellaceae. Those CYPs could not be assigned to any of the already defined CYP (sub)families and were therefore treated as new (sub)families. A total of five new families were detected in both carton strains, while the number of subfamilies was 42 (in *I. lentus*) and 44 (in *I. delicatus*). These numbers remain lower than those observed in other Chaetothyriales, mainly Herpotrichiellaceae, where they range from 65 to 170.

**Table 4:** Distribution of CYP p450 genes in in ant-associated fungi and other Chaetothyriales fungi

Species	Ecology	Genes of family	Genes of new family	Genes of total family	Genes of subfamily	Genes of new subfamily	Total genes
**Trichomeriaceae**							
*Incumbomyces lentus*	Carton	58	5	63	15	42	105
*Incumbomyces delicatus*	Carton	62	5	67	17	44	111
*Arthrocladium fulminans*	Patient	66	6	72	20	45	117
**Domatia**							
Chaetothyriales sp. CBS 135597	Domatia	74	9	83	27	56	139
Chaetothyriales sp. CBS 134916	Domatia	68	6	74	19	54	128
**Cyphellophoraceae**							
*Cyphellophora attinorum*	Ant	116	9	125	26	88	213
**Herpotrichiellaceae**							
*Exophiala xenobiotica*	Oil sludge	142	18	160	33	108	268
*Exophiala oligosperma*	Patient	116	20	136	28	94	230
*Rhinocladiella mackenziei*	Patient	135	25	160	35	109	269
*Capronia coronata*	Wood	81	9	90	29	61	151
*Cladophialophora psammophila*	Polluted soil	178	18	196	44	126	322
*Cladophialophora bantiana*	Patient	139	16	155	35	99	254
*Fonseceae pedrosoi*	Patient	142	11	153	49	44	197

The CYPs families of carton and domatia species are very similar (Additional file 4: Table S4). One of the main differences concerns CYP 6001, which is present in carton fungi but absent from domatia fungi. The CYPs are also close to those of *Arthrocladium fulminans,* a member of the same family as *Incumbomyces*, Trichomeriaceae, and solely known from an infection in a human patient. *A. fulminans* has four more CYP families (CYP5199, CYP570, CYP619, CYP60) compared to domatia species; the remaining CYPs are shared. Seven CYPs (CYP5076, CYP5077, CYP5081, CYP5282, CYP5295, CYP5307, CYP631) were found only in domatia and were lacking in other members of Chaetothyriales. Most of these genes are related to xenobiotic or secondary metabolism (Chadha et al. 2018).

### Secondary metabolism

**Fig. 3:**
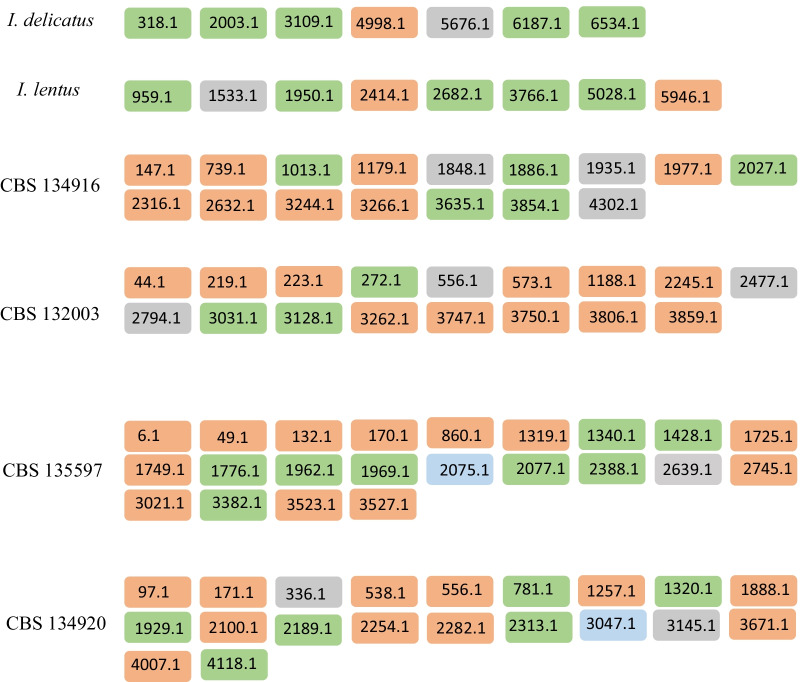
The region and of amount of secondary-metabolite gene classes in ant-associated fungi

Secondary metabolism refers to the metabolic pathways and small molecular products involved in ecological interaction, which are not essential for the survival of the organism but may be significant, e.g. for the colonization of a specific niche. Summarizing the wide diversity of secondary metabolites, the following broad types can be distinguished according to enzymes involved in synthetic pathways: polyketides (PKS), non-ribosomal peptides (NRPS), and terpenes and indole alkaloids (Keller et al. 2005). Several of the sequenced fungal genomes contained hybrid genes, such as PKS-NRPS. *Incumbomyces delicatus* and *I. lentus* possess 7 and 8 biosynthetic clusters, respectively (Fig. 3, Table 5). The NRPS type is prevalent in clusters of both carton species. The type III PKS cluster (t3 PKS), previously reported in Herpotrichiellaceae, Cyphellophoraceae, and Trichomeriaceae is absent from carton and domatia species. In addition, both species had only one or two I PKS, which is far less than observed in domatia strains (11 on average). The number of terpenes in *Incumbomyces* was also far less than in remaining Chaetothyriales (Teixeira et al. 2017).

**Table 5:** Summary of secondary-metabolite gene classes in ant-associated fungi

Species	Terpene	III PKS	I PKS	III PKS/I PKS	NRPS	I PKS/NRPS
Incumbomyces lentus	1	0	2	0	5	0
Incumbomyces delicatus	1	0	1	0	5	0
Chaetothyriales sp CBS 134916	3	0	8	0	5	0
Chaetothyriales sp CBS 132003	3	0	11	0	3	0
Chaetothyriales sp CBS 134920	2	0	11	0	6	1
Chaetothyriales sp CBS 135597	1	0	12	0	8	1

### Asexual and sexual reproduction

**Fig. 4:**
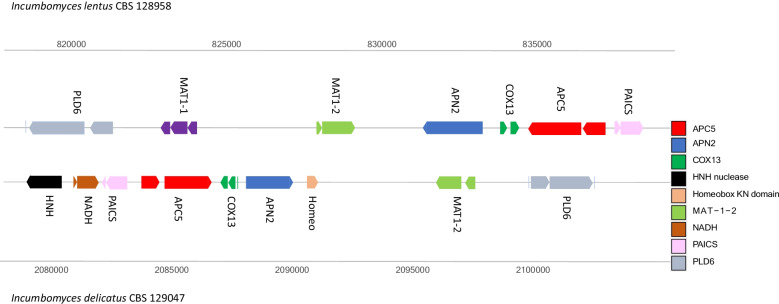
Mating type locus structure and flanking regions of two carton species

The mating type locus is a unique region in the fungal genome, playing a central role in the sexual cycle. We identified the mating type idiomorph, flanking genes and heterokaryon incompatibility protein (HET-PF06985) in the genomes of *Incumbomyces* carton fungi and compared these with four domatia species and other members of Chaetothyriales described previously. In none of the *Incumbomyces* species, sporulation has been observed, neither sexual nor clonal (Quan et al. 2020a, b). *I. delicatus* (CBS 129047) contained a single mating type (*MAT1-2*) and thus was found to be heterothallic. In contrast, *I. lentus* (CBS 128958) was confirmed to have both *MAT1-1* and *MAT1-2*, which were closely clustered in a single assembled scaffold; hence *Incumbomyces lentus* was homothallic (Fig. 4).

We confirmed the *MAT* flanking genes *APN2, APC5, SLA2* and *COX13*, which are highly conserved and are distributed widely in members of Eurotiomycetes (Coppin et al. 1997; Fraser et al. 2007; Paoletti et al. 2007). Nevertheless, significant differences in the flanking structure were observed between the two *Incumbomyces* species. In *I. lentus* CBS 128958, the *APN2, COX13*, and *APC5* genes were in synteny in the right flanking region, while in *I. delicatus* CBS 129047 they were in the left flanking region, in inversed order. The *SLA2* genes of these two species were not close to other flanking genes. Concerning *I. lentus*, the *MAT* locus is localized in scaffold 5 (*SLA2* in scaffold 2). In *I. delicatus*, the *MAT* locus is localized in scaffold 8 (*SLA2* in scaffold 6). Both carton species have the *COX13* genes, which are absent from most Chaetothyriales but present in domatia fungi and in members of Trichomeriaceae, i.e. in *Incumbomyces* and in *A. fulminans.* The heterokaryon incompatibility protein (*HET*) ranged from 73 in *I. lentus* to 105 in *I. delicatus*. In contrast, the number in domatia strains ranged from 10 in CBS 134920 to 31 in CBS 134916. In other species of Chaetothyriales, the number was extremely variable, ranging from 1 to 134 (Additional file 5: Table S5).

### Enzymes representing specific types of ecology

To better understand the ecology of carton fungi and other Chaetothyriales, we selected five types of enzymes, representing specific types of ecology, and listed the number of genes in each category, such as cellulases and pectinases (plant-associated compounds), lipases (animal-associated compounds), chitinases (potentially involved in decomposition of chitinous ant bodies), and ligninolytic enzymes (involved in wood degradation). An observation which is difficult to explain is that *A. fulminans*, only known from human infection, is unique in Chaetothyriales by having 12 pectinase genes (Table 6).

**Table 6:** The number of five types of enzymes, representing specific types of ecology in each category

Species	Strain number	Cellulases	Lipases	Chitinases	Ligininolytic	Pectinases
*Incumbomyces lentus*	CBS 128958	7	22	3	8	0
*Incumbomyces delicatus*	CBS 129047	8	20	5	8	0
*Arthrocladium fulminans*	CBS 136243	11	44	28	33	12
*Chaetothyriales sp.*	CBS 135597	9	16	10	7	0
*Chaetothyriales sp.*	CBS 134916	11	15	5	9	0
*Phialophora attinorum*	CBS 131958	15	53	6	9	0
*Phialophora europaea*	CBS 101466	17	32	7	11	0
*Exophiala dermatitidis*	NIH 8656	9	21	5	7	0
*Exophiala sideris*	CBS 121828	11	27	5	7	0
*Exophiala xenobiotica*	CBS 118157	13	34	7	10	0
*Exophiala oligosperma*	CBS 725.88	8	36	6	10	0
*Exophiala spinifera*	CBS 89968	8	43	5	9	0
*Exophiala aquamarina*	CBS 119918	13	50	6	7	0
*Exophiala mesophila*	CBS 402.95	11	16	4	9	0
*Rhinocladiella mackenziei*	CBS 650.93	11	27	2	6	0
*Capronia coronata*	CBS 617.96	11	21	5	8	0
*Cladophialophora psammophila*	CBS 110553	12	40	4	12	0
*Cladophialophora bantiana*	CBS 173.52	11	35	4	12	0
*Fonsecaea multimorphosa*	CBS 102226	12	31	3	10	0
*Cladophialophora immunda*	CBS 834.96	11	24	3	13	0
*Fonsecaea pedrosoi*	CBS 271.37	10	29	4	11	0
*Cladophialophora yegresii*	CBS 114405	14	28	3	9	0
*Cladophialophora carrionii*	CBS 160.54	13	30	3	8	0
*Phialophora americana*	CBS 27337	14	38	3	8	0

Cellulases representing the ecology related to plant-associated compounds, lipases representing the ecology related to animal-associated compounds, chitinases potentially involved in decomposition of chitinous ant bodies, ligninolytic enzymes involved in wood degradation

## Discussion

The genus *Incumbomyces*, containing two described species isolated from walls of ant-made carton nests (Quan et al. 2021), clusters phylogenetically among members of Trichomeriaceae, as a sister group of *Bradymyces*. It may be questioned whether the genus shows highest similarity with its family members due to shared phylogeny, or with ant-associated Chaetothyriales sharing a similar habitat. The only member of the Trichomeriaceae for which a genome has been sequenced is *Arthrocladium fulminans* (Moreno et al. 2020). This species is rather exceptional in Trichomeriaceae, being known from two isolates that caused severe infections in humans (Diallo et al. 2017; Egenlauf et al. 2019), while most members of the family comprise surface-colonizing ‘sooty moulds’ (Chomnunti et al. 2012). In addition to this life style, a significant number of ant-associated species—both carton- and domatia-associated—clustered in the family (Fig. 1). Another group combining oligotrophic surface colonization with pathogenicity is *Bradymyces*, where an infection in fish was described (Hubka et al. 2014). An interesting analogy of oligotrophy and infectious ability was published by Moreno et al. (2019), hypothesizing an environmental habitat of the neuroinvasive species *Rhinocladiella mackenziei* in the hydrocarbon-polluted desert. The expanded cytochromes may be instrumental in these seemingly very different life styles. In addition to cytochromes, the species also had choline permease which assists in conversion to the osmoprotectant glycine betaine, enhancing survival in the desert. Numerous members of Chaetothyriales, particularly those in Herpotrichiellaceae and scattered in other families, are environmental but predisposed for infection by their melanin, extremotolerance and hydrocarbon assimilation, being pronounced opportunists. The evolution of genes involved in colonization of these habitats deserves attention. Ant domatia are rich in low-molecular volatiles with low toxicity (Prenafeta et al. 2021), which may have been an evolutionary starting point, but more data are needed from comparable species in Trichomeriaceae.

For some general genomic characters, such as genome size, GC content, and gene number, the carton fungi in *Incumbomyces* proved to be similar to *A. fulminans*. The domatia fungi (“domata-group” in Fig. 1) differ significantly from all remaining Chaetothyriales known thus far by having small genomes containing a limited number of genes (Moreno et al. 2019; Table 1). The GH families i.e. GH27, GH36, GH79, and GH92 are mostly linked to decomposition of polysaccharides in plant cell walls. Ant nests and tunnels inhabited by *Incumbomyces* species consist of wood pulp that has been chewed by the ants, whereby the fungal hyphae enhance constructive strength. A CAZy difference between carton and domatia concerns AA7 enzymes in AA class. The amount of these enzymes in carton is much more than that in domatia. Known AA7 enzymes are potentially involved in the biotransformation or detoxification of lignocellulosic compounds (Levasseur et al. 2013). Carton also consists of ant-chewed wood and other plant material, while this material is absent from domatia. Indeed, the total number of CAZymes in carton fungi is higher than that in domatia fungi, indicating that carton fungi have more enzymes to decompose plant material and to synthesize fungal cell components. The habitat of ant debris inside domatia apparently does not require these enzymes.

Ants secrete pheromones such as low-molecular hydrocarbons and ketones (David Morgan 2009) to protect the brood against fungal and bacterial pathogens. Many fungi can be isolated from the nests as their propagules are easily trapped in the complex networks, but few are able to grow in this environment. However, members of Chaetothyriales seem relatively resistant to these compounds, allowing them to colonize the somewhat toxic ant constructions (Mayer et al. 2018). Teixeira et al. (2017) observed cytochrome (CYP) family expansion in Chaetothyriales. The authors actually only analyzed members of Herpotrichiellaceae, a family with a strong association with domestic environments polluted with aromatic hydrocarbons (Isola et al. 2013). CYP expansion is consistent with this habitat. For example, CYP 530 is thought to participate in the degradation of several fatty acids and hydrocarbons. It was found with 12 copies in *Cladophialophora psammophila,* a species known from a single isolate from hydrocarbon-polluted soil. This CYP was not found in any of the carton or domatia strains, suggesting that the fungi, reside in a lower-toxic habitat. This absence may be evolutionarily ancestral, as chaetothyrialean members in the derived family Herpotrichiellaceae that survive in hydrocarbon-polluted domesticated environments have shown significant expansion of CYP genes (Teixeira et al. 2017).

Very large differences related to secondary metabolism were noted between species, even among species sharing a similar ecology. Carton and domatia fungi are associated to ants, the enzymes related to the secondary metabolism of both fungi are very different. Particularly the differences in I PKS and NRPS are significant (Fig. 3). The exact ecological meaning of these differences is as yet unclear and needs comparison with additional members of Trichomeriaceae from other habitats.

Similar to most species of Trichomeriaceae, sporulation in vitro is nearly absent from cultured *Incumbomyces* species. Only a few species are known by their *Trichomerium* sexual states in the natural habitat (Chomnunti et al. 2012). *Incumbomyces lentus* has both *MAT1-1* and *MAT1-2*, a condition thus far only proven in sexual *Capronia* species (Teixeira et al. 2017). The species may thus have a homothallic sexual cycle in nature. The other carton species, *I. delicatus* only has a single *MAT* gene, with a structure similar with domatia species and *A. fulminans*. Among the known species of Chaetothyriales, only those isolated via their sexual state in nature (*Capronia semimmersa, C. epimyces* and *C. coronata*) have been proven to harbor two mating type genes, while all asexual species described to date have only a single mating type gene (Teixeira et al. 2017). Also the four domatia strains and *Arthrocladium fulminans* in Trichomeriaceae harbored only a single *MAT* gene (Moreno et al. 2019). The *MAT* locus of the two species of *Incumbomyces* is remarkably different, with a large translocation in *I. lentus* where *APN2*, *COX13* and *APC5* are in the right flanking region. In *I delicatus* they are in the left flanking region as in *Arthrocladium* and domatia fungi, but in different order. In general, the *MAT* location is extremely variable in Chaetothyriales (Teixeira et al. 2017) for reasons which have not been clarified.

## Supplementary Information

**Additional file 1: Table S1**. Basic information of Chaetothyriales species included in the study.

**Additional file 2: Table S2**. The GenBank data of strains in this study.

**Additional file 3: Table S3**. Specific genes of CAZymes in ant-associated fungi and other Chaetothyriales fungi.

**Additional file 4: Table S4**. Specific genes of Cytochrome P450 in ant-associated fungi and other Chaetothyriales fungi.

**Additional file 5: Table S5**. The numbers of Heterokaryon incompatibility protein (HET-PF06985) in Chaetothyriales.

## Data Availability

The datasets generated for this study can be found in the GenBank Accessions: JACJVS000000000 (*I. delicatus*) and JACJVT000000000 (*I. lentus*).
