# Supplementary material for: Black fungi and ants: a genomic comparison of species inhabiting carton nests versus domatia
Source: IMA Fungus. 2022 Mar 7;13:4. doi: 10.1186/s43008-022-00091-5 (PMC8900376; doi:10.1186/s43008-022-00091-5)
Supplement: Supplementary file 2 — Additional file 2: Table S2. The GenBank data of strains in this study. [file 43008_2022_91_MOESM2_ESM.docx]

Table S2. Strain GenBank data with proximate ecology per species.

| **Clade** | **Species** | **Accession number** | **ITS** | **LSU** |
| --- | --- | --- | --- | --- |
| Herpotrichiellaceae | *Exophiala spinifera* | D22I | MH010942.1 | MH012097.1 |
|  | *Rhinocladiella similis* | PW3041 | LC158611.1 | LC158635.1 |
|  | *Exophiala exophialae* | CBS 668.76 (T) | AY156973.1 | NG_059252.1 |
|  | *Exophiala nigra* | CBS 535.94 (T) | KY115191.1 | NG_059253.1 |
|  | *Exophiala oligosperma* | CBS 127587 | MH864631.1 | MH876068.1 |
|  | *Exophiala polymorpha* | CBS 138920 (T) | KP070763.1 | NG_059237.1 |
|  | *Exophiala italica* | MFLUCC 16-0245 | KY496744.1 | KY496723.1 |
|  | *Thysanorea aquatica* | MFLCC 15-0966 | MG922572.1 | MG922576.1 |
|  | *Thysanorea papuana* | CBS 212.96 (T) | MH862572.1 | MH874198.1 |
|  | *Capronia kleinmondensis* | CBS 122671 (T) | MH863226.1 | EU552107.1 |
|  | *Rhinocladiella atrovirens* | CBS 264.49 (T) | MH856518.1 | EU041869.1 |
|  | *Exophiala dermatitidis* | CBS 207.35 (T) | MH855649.1 | NG_059225.1 |
|  | *Capronia mansonii* | CBS 101.67 (T) | AF050247.1 | AY004338.1 |
|  | *Capronia munkii* | CBS 615.96 (T) | MH862601.1 | EF413604.1 |
|  | *Exophiala hongkongensis* | HKU 32 (T) | JN625231.2 | NG_059264.1 |
|  | *Capronia dactylotricha* | CBS 604.96 (T) | AF050243.1 | KX712343.1 |
|  | *Capronia pilosella* | AFTOL-ID 657 | DQ826737.1 | DQ823099.1 |
|  | *Veronaea compacta* | CBS 268.75 (T) | EU041819.1 | NG_057790.1 |
|  | *Exophiala brunnea* | CBS 587.66 (T) | MH858890.1 | KX712342.1 |
|  | *Exophiala jeanselmei* | CBS 507.90 (T) | AY156963.1 | KJ930161.1 |
|  | *Exophiala moniliae* | CBS 520.76 (T) | KF881967.1 | KJ930162.1 |
|  | *Exophiala bergeri* | CBS 353.52 (T) | MH857080.1 | NG_059199.1 |
|  | *Exophiala sideris* | D88 | KC315801.1 | HM627072.1 |
|  | *Capronia fungicola* | CBS 614.96 (T) | KY484990.1 | NG_058761.1 |
|  | *Capronia nigerrima* | CBS 513.69 | MH859363.1 | AY605075.1 |
|  | *Phaeoannellomyces elegans* | CBS 101597 | NR_155687.1 | KY115194.1 |
|  | *Exophiala psychrophila* | CBS 191.87 (T) | NR_145371.1 | MH873750.1 |
|  | *Exophiala lignicola* | CBS 144622 (T) | NR_163358.1 | NG_066324.1 |
|  | *Exophiala nidicola* | FMR 3889 (T) | NR_161045.1 | MG701056.1 |
|  | *Exophiala nishimurae* | CBS 101538 (T) | NR_137092.1 | KX712351.1 |
|  | *Exophiala palmae* | UPCB 86822 (T) | NR_158414.1 | NG_064428.1 |
|  | *Exophiala heteromorpha* | CBS 232.33 (T) | NR_111184.1 | NG_063975.1 |
|  | *Exophiala eucalypticola* | CBS 143412 (T) | MH107891.1 | NG_063955.1 |
|  | *Exophiala eucalypti* | CPC 27630 | KY173411.1 | KY173502.1 |
|  | *Capronia camelliae-yunnanensis* | CGMCC 3.19061 (T) | NR_164589.1 | NG_066425.1 |
|  | *Capronia leucadendri* | CBS 122672 (T) | NR_156212.1 | MH874754.1 |
|  | *Capronia parasitica* | CBS 123.88 | AF050252.1 | FJ358225.1 |
|  | *Rhinocladiella quercus* | CPC 26621 (T) | KX306769.1 | NG_059698.1 |
|  | *Exophiala attenuata* | F10685 | KT013095.1 | KT013094.1 |
|  | *Exophiala lecanii-comi* | CBS 12333 (T) | MH855383.1 | NG_059200.1 |
|  | *Exophiala castellanii* | CBS 158.58 (T) | MH857734.1 | KF928522.1 |

Table 1. Continued

| **Clade** | **Species** | **Accession number** | **ITS** | **LSU** |
| --- | --- | --- | --- | --- |
|  | *Exophiala mesophila* | CBS 402.95 | MH862536.1 | KX712349.1 |
|  | *Chaetothyriales sp.* | T210 | KF614880 | KF614880 |
|  | *Rhinocladiella phaeophora* | CBS 496.78 (T) | EU041811.1 | NG_057785.1 |
|  | *Rhinocladiella aquaspersa* | CBS 122635 | GU017732.1 | KX822357.1 |
|  | *Rhinocladiella tropicalis* | RA776 | KU854928.1 | KX356663.1 |
|  | *Exophiala pisciphila* | CBS 537.73 (T) | DQ826739.1 | NR121269 |
|  | *Exophiala bonariae* | CCFEE 5792 (T) | JX681046.1 | KR781083.1 |
|  | *Exophiala opportunisticica* | CBS 122268 | KF928436.1 | KF928500.1 |
|  | *Exophiala cancerae* | CBS 115142 | MH862980.1 | MH874540.1 |
|  | *Exophiala abietophila* | CBS 145038 (T) | NR_163357.1 | NG_066323.1 |
|  | *Veronaea constricta* | CBS 572.90 | MH862237.1 | MH873920.1 |
|  | *Veronaea japonica* | CBS 776.83 (T) | MH861692.1 | NG_057789.1 |
|  | *Veronaea botryosa* | CBS 254.57 (T) | MH857711.1 | MH869255.1 |
|  | *Chaetothyriales sp.* | CBS 128956 | KX822529 | KX822529 |
|  | *Exophiala crusticola* | CBS 119970 (T) | MH863070.1 | NG_059220.1 |
|  | *Minimelanolocus obscurus* | MFLUCC 15-0416 | KR215606.1 | KR215611.1 |
|  | *Minimelanolocus melanicus* | MFLUCC 15-0415 (T) | KR215608.1 | KR215613.1 |
|  | *Minimelanolocus asiaticus* | MFLUCC 15-0237 (T) | NR_154179.1 | KR215610.1 |
|  | *Minimelanolocus curvatus* | MFLUCC 15-0259 (T) | KR215605.1 | KR215609.1 |
|  | *Minimelanolocus aquaticus* | MFLUCC 15-0414 (T) | KR215607.1 | KR215612.1 |
|  | *Capronia acutiseta* | CBS 618.96 (T) | NR_154744.1 | NG_058859.1 |
|  | *Chaetothyriales sp.* | CBS 129051 | KX822540 | KX822540 |
|  | *Rhinocladiella anceps* | AFTOL ID 659 | DQ826740.1 | DQ823102.1 |
|  | *Melanoctona tectonae* | MFLUCC 12-0389 (T) | KX258778.1 | KX258779.1 |
|  | *Chaetothyriales sp.* | CBS 129050 | KX822532.1 | KX822532 |
|  | *Capronia coronata* | ATCC 56201 (T) | NR_154745.1 | AF050242.1 |
|  | *Exophiala angulospora* | CBS 482.92 (T) | MH862370.1 | KF155190.1 |
|  | *Rhinocladiella mackenziei* | CBS 368.92 | MH862361.1 | EU041866.1 |
|  | *Rhinocladiella coryli* | CPC 26654 (T) | KX306768.1 | KX306793.1 |
|  | *Chaetothyriales sp.* | T171 | KF614875 | KF614875 |
|  | *Chaetothyriales sp.* | T22 | KF614881 | KF614881 |
|  | *Exophiala alcalophila* | CBS 520.82 (T) | MH861524.1 | NG_059189.1 |
|  | *Fonsecaea minima* | CBS 125757 (T) | MH863743.1 | KF928520.1 |
|  | *Fonsecaea pugnacius* | CBS 139214 (T) | NR_155089.1 | NG_058177.1 |
|  | *Fonsecaea pedrosoi* | CBS 271.37 (T) | AB114127.1 | KJ930166.1 |
|  | *Fonsecaea erecta* | CBS 125763 | KC886414.1 | KF155186.1 |
|  | *Fonsecaea brasiliensis* | BMU 07620 | KJ701015.1 | KJ930163.1 |
|  | *Cladophialophora devriesii* | CBS 147.84 (T) | EU103985.1 | KC809989.1 |
|  | *Cladophialophora immunda* | CBS 834.96 (T) | MH862619.1 | KC809990.1 |
|  | *Chaetothyriales sp.* | T367 | KF614894 | KF614894 |
|  | *Cladophialophora arxii* | CBS 306.94 (T) | EU103986.1 | NG_058959.1 |
|  | *Cladophialophora minourae* | CBS 556.83 (T) | AY251087.1 | NG_058763.1 |

Table1 Continued

| **Clade** | **Species** | **Accession number** | **ITS** | **LSU** |
| --- | --- | --- | --- | --- |
|  | *Cyphellophora laciniata* | CBS 190.61 (T) | EU035416.1 | KF928547.1 |
|  | *Cyphellophora suttonii* | CBS 449.91 (T) | KC455243.1 | KC455256.1 |
|  | *Cyphellophora fusarioides* | CBS 130291 (T) | MH865596.1 | JQ766486.1 |
|  | *Cyphellophora pauciseptata* | CBS 284.85 (T) | JQ766438.1 | JQ766519.1 |
|  | *Cyphellophora sessilis* | CBS 243.85 (T) | AY857542.1 | EU514700.1 |
|  | *Cyphellophora europaea* | CBS 101466 (T) | KF928473.1 | KC455259.1 |
|  | *Cyphellophora vermispora* | CBS 228.86 (T) | MH861947.1 | KC455257.1 |
|  | *Cyphellophora reptans* | CBS 113.85 (T) | EU514699.1 | EU514699.1 |
|  | *Chaetothyriales sp.* | CBS 128959 | KX822542 | KX822542 |
|  | *Phialophora capiguarae* | CBS 131954 | KF928465.1 | KF928529.1 |
|  | *Cyphellophora clematidis* | CBS 144983 | MK442577.1 | MK442519.1 |
|  | *Chaetothyriales sp.* | Cecr4 | KX822476 | KX822476 |
|  | *Chaetothyriales sp.* | CBS 135086 | KX822336 | KX822336 |
|  | *Chaetothyriales sp.* | Trii4 | KX822551 | KX822551 |
|  | *Chaetothyriales sp.* | CBS 134920 | KX822324 | KX822324 |
|  | *Chaetothyriales sp.* | CBS 134916 | KX822344 | KX822344 |
|  | *Chaetothyriales sp.* | CBS 128963 | KX822328 | KX822328 |
|  | *Chaetothyriales sp.* | CBS 128966 | KX822331 | KX822331 |
|  | *Chaetothyriales sp.* | CBS 128973 | KX822354 | KX822354 |
|  | *Chaetothyriales sp.* | CBS134923 | KX822319 | KX822319 |
| Trichomeriaceae | *Anthracinomyces petraeus* | CGMCC 3.17315 | KP174843.1 | KP174924.1 |
|  | *Anthracinomyces ramosus* | CGMCC 3.16367 | KP174846.1 | KP174922.1 |
|  | *Knufia perforans* | CBS 885.95 (T) | MH862564.1 | NG_042586.1 |
|  | *Knufia petricola* | CBS 726.95 (T) | MH862556.1 | NG_042775.1 |
|  | *Knufia vaticanii* | CCFEE 5939 (T) | KP791780.1 | KR781068.1 |
|  | *Knufia marmoricola* | CCFEE 6201 | KP791790.1 | KR781077.1 |
|  | *Knufia karalitana* | CCFEE 5921 | KP791784.1 | KR781072.1 |
|  | *Knufia epidermidis* | CBS 120353 (T) | NR_111330.1 | NG_042475.1 |
|  | *Knufia cryptophialidica* | DAOM 216555 (T) | JN040501.1 | JN040500.1 |
|  | *Knufia mediterranea* | CBS 139721 (T) | KP791794.1 | KR781081.1 |
|  | *Chaetothyriales sp.* | T261 | KF614886.1 | KF614886 |
|  | *Arthrocladium tropicale* | CBS 134926 (T) | KX822543.1 | NG_057119.1 |
|  | *Arthrocladium tardum* | CBS 127021 (T) | KT337440.1 | NG_057089.1 |
|  | *Arthrocladium caudatum* | CBS 457.67 (T) | MH859032.1 | NG_057084 |
|  | *Arthrocladium fulminans* | CBS 136243 (T) | KT337439.1 | NG_057088.1 |
|  | *Incumbomyces lentus.* | CBS 128958 | KX822541 | KX822541 |
|  | *Chaetothyriales sp.* | CBS 129049 | KX822531 | KX822531 |
|  | *Incumbomyces delicatus* | CBS 129047 | KX822533.1 | KX822533 |
|  | *Exophiala placitae* | CBS 121716 (T) | MH863143.1 | MH874694.1 |
|  | *Chaetothyriales sp.* | T179 | KF614876.1 | KF614876.1 |

Table 1. Continued

| **Clade** | **Species** | **Accession number** | **ITS** | **LSU** |
| --- | --- | --- | --- | --- |
|  | *Cladophialophora eucalypti* | CBS 145551 (T) | MK876380.1 | MK876419.1 |
|  | *Cladophialophora pucciniophila* | KUS F23645 | JF263533.1 | JF263534.1 |
|  | *Cladophialophora proteae* | CBS 111667 (T) | EU035411.1 | EU035411.1 |
|  | *Strelitziana albiziae* | CBS 126497 (T) | MH864122.1 | HQ599585.1 |
|  | *Strelitziana eucalypti* | CBS 128214 | HQ599596.1 | HQ599597.1 |
|  | *Strelitziana australiensis* | CBS 124778 (T) | GQ303295.1 | GQ303326.2 |
|  | *Strelitziana cliviae* | CPC 19822 (T) | KC005772.1 | NG_042750.1 |
|  | *Bradymyces alpinus* | CCFEE 5493 (T) | HG793052.1 | GU250396.1 |
|  | *Bradymyces graniticola* | F6A | KX179910.1 | KX179912.1 |
|  | *Bradymyces oncorhynchi* | CCF 4369 (T) | NR_132843.1 | NG_058643.1 |
|  | *Chaetothyriales sp.* | T333 | KF614873.1 | KF614873.1 |
|  | *Trichomerium foliicola* | MFLUCC 10-0078 (T) | JX313655.1 | JX313661.1 |
|  | *Trichomerium gloeosporum* | MFLUCC 10-0087 (T) | JX313656.1 | JX313662.1 |
|  | *Trichomerium dioscoreae* | CBS 138870 (T) | NR_137946.1 | NG_058126.1 |
|  | *Trichomerium deniquelatum* | MFLUCC 10-0884 (T) | JX313654.1 | JX313660.1 |
|  | *Trichomerium eucalypti* | CBS 143443 (T) | NR_156672.1 | NG_058525.1 |
|  | *Chaetothyriales sp.* | T13 | KF614778 | KF614778 |
|  | *Chaetothyriales sp.* | T9 | KF614780.1 | KF614780.1 |
|  | *Chaetothyriales sp.* | CBS 128943 | KX822485.1 | KX822485 |
|  | *Chaetothyriales sp.* | CBS 129046 | KX822526.1 | KX822526 |
|  | *Knufia peltigerae* | CGMCC 3.17283 | KP174864.1 | KP174935.1 |
|  | *Knufia tsunedae* | FMR 10621 (T) | NR_132842.1 | HG003672.1 |
|  | *Metulocladosporiella musicola* | CBS 110960 (T) | MH862870.1 | DQ008153.1 |
|  | *Brycekendrickomyces acaciae* | CBS 124104 (T) | NR_132828.1 | NG_058633.1 |
|  | *Exophiala encephalarti* | CBS 128210 | HQ599588.1 | HQ599589.1 |
|  | *Ceramothyrium melastoma* | CPC 19837 (T) | NR_111822.1 | NG_042749.1 |
|  | *Neostrelitziana acaciigena* | CBS 139903（T） | NR_137987.1 | NG_058165.1 |
|  | *Strelitziana africana* | CBS 120037 | DQ885895.1 | DQ885895.1 |
|  | *Arthrophiala arthrospora* | COAD 658 | KY173473.1 | KX447143.1 |
|  | *Lithohypha aloicola* | CPC 35996(T) | NR_166313.1 | MN567611.1 |
| Chaetothyrialceae | *Ceramothyrium exiguum* | VTCCF-1209 (T) | LC360297.1 | LC360295.1 |
|  | *Nullicamyces eucalypti* | CPC 32942 (T) | MH327807.1 | NG_064546.1 |
|  | *Ceramothyrium aquaticum* | VTCCF-1210 (T) | LC360299.1 | LC360296.1 |
|  | *Ceramothyrium phuquocense* | VTCCF-1206 (T) | LC360298.1 | LC360294.1 |
|  | *Camptophora schimae* | IFRDCC 2664 | MF285231.1 | MF285233.1 |
|  | *Camptophora hylomeconis* | CBS 113311 (T) | EU035415.1 | EU035415.1 |
|  | *Aphanophora eugeniae* | CBS 124105 (T) | FJ839617.1 | NG_056965.1 |
|  | *Phaeosaccardinula dendrocalami* | IFRDCC 2649 (T) | NR_137820.1 | NG_060116.1 |
|  | *Phaeosaccardinula multiseptata* | IFRDCC 2639 (T) | NR_132894.1 | KF667244.1 |
|  | *Phaeosaccardinula ficus* | MFLUCC 10-0009 (T) | HQ895840.1 | NG_059455.1 |
|  | *Ceramothyrium menglunense* | MFLUCC 16-1874 (T) | KX524148.1 | KX524146.1 |

Table 1 Continued

| **Clade** | **Species** | **Accession number** | **ITS** | **LSU** |
| --- | --- | --- | --- | --- |
|  | *Chaetothyrium brischoficola* | MFLUCC 10-0083 (T) | HQ895839.1 | HQ895836.1 |
|  | *Vonarxia vagans* | CBS 123533 (T) | FJ839636.1 | NG_057821.1 |
|  | *Chaetothyrium agathis* | MFLUCC 12-0113 (T) | KP744437.1 | KP744480.1 |
|  | *Ceramothyrium podocarpi* | CPC 19826 (T) | KC005773.1 | NG_042751.1 |
|  | *Fumagopsis stellae* | CBS 145078 (T) | NR_161138.1 | NG_066293.1 |
|  | *Ceramothyrium carniolicum* | CBS 175.95 | KC978733.1 | KC455251.1 |
|  | *Ceramothyrium thailandicum* | MFLUCC 10-0008 (T) | HQ895838.1 | NG_058817.1 |
|  | *Ceramothyrium ficus* | MFLUCC 15-0228 (T) | KT588601.1 | NG_058927.1 |
|  | *C. longivolcaniforme* | MFLUCC 16-1306 (T) | KP324929.1 | NG_058904.1 |
| Epibryaceae | *Cladophialophora humicola* | CBS 117536 (T) | EU035408.1 | NG_058850.1 |
|  | *Cladophialophora minutissima* | CBS 121758 (T) | MH863155.1 | NG_058851.1 |
|  | *Cladophialophora sylvestris* | CBS 35083 (T) | EU035413.1 | EU035413.1 |
|  | *Epibryon bryophilum* | CBS 126278 | MH863955.1 | MH875414.1 |
|  | *Epibryon interlamellare* | CBS 126286 | MH863958.1 | MH875417.1 |
|  | *Epibryon turfosorum* | CBS 126587 | MH864165.1 | MH875627.1 |
|  | *Chaetothyriales sp.* | L1992 | KT263083.1 | KT263083.1 |
|  | *Chaetothyriales sp.* | L1993 | KT263084.1 | KT263084.1 |
|  | *Chaetothyriales sp.* | L1994 | KT263085.1 | KT263085.1 |
|  | *Lichenodiplis lecanorae* | L | -- | KT285909.1 |
| Outgroup  Melanina | *Chaetothyriales sp.* | A581 | MT193582 | KT263163.1 |
|  | *Chaetothyriales sp.* | A933 | MT193581 | KT270641 |
|  | *Chaetothyriales sp.* | A872 | MT193584 | KT270601 |
|  | *Chaetothyriales sp.* | A957 | MT193583 | KT270659 |
|  |  |  |  |  |
